# Chromosome-Level Genome Assembly of *Bupleurum chinense* DC Provides Insights Into the Saikosaponin Biosynthesis

**DOI:** 10.3389/fgene.2022.878431

**Published:** 2022-03-31

**Authors:** Quanfang Zhang, Min Li, Xueyan Chen, Guoxia Liu, Zhe Zhang, Qingqing Tan, Yue Hu, Yangyang Fan, Yanyan Liu, Tongshan Zhu, Xue Yang, Mingming Yue, Xun Bu, Yongqing Zhang

**Affiliations:** ^1^ Institute of Crop Germplasm Resources, Shandong Academy of Agricultural Sciences, Jinan, China; ^2^ Shandong University of Traditional Chinese Medicine, Jinan, China

**Keywords:** Bupleurum chinense, saikosaponin biosynthesis, genome assembly, chromosome-level genome, evolution, haplotype, gene families

## Abstract

*Bupleurum chinense* DC is a plant widely used in Chinese traditional medicine. Saikosaponins are the major bioactive constituents of *B. chinense* DC. Saikosaponins biosynthesis in *Bupleurum* has been more intensively studied than any other metabolic processes or bioactive constituents. However, whole-genome sequencing and chromosome-level assembly for *Bupleurum* genus have not been reported yet. Here, we report a high-quality chromosome-level genome of *B. chinense* DC. through the integration of PacBio long-read sequencing, Illumina short-read sequencing, and Hi-C sequencing. The genome was phased into haplotype 0 (621.27 Mb with a contig N50 of 16.86 Mb and a scaffold N50 of 92.25 Mb) and haplotype 1 (600.48 Mb with a contig N50 of 23.90 Mb and a scaffold N50 of 102.68 Mb). A total of 45,909 and 35,805 protein-coding genes were predicted in haplotypes 0 and 1, respectively. The enrichment analyses suggested that the gene families that expanded during the evolution of *B. chinense* DC are involved in the biosynthesis of isoquinoline alkaloid, tyrosine, and anthocyanin. Furthermore, we analyzed the genes involved in saikosaponin biosynthesis and determined the candidate P450 and UGT genes in the third stage of saikosaponins biosynthetic, which provided new insight into the saikosaponins biosynthetic. The genomic data provide a valuable resource for future investigations of the molecular mechanisms, biological functions, and evolutionary adaptations of *B. chinense* DC.

## 1 Introduction

*Bupleuri radix* (Chaihu) is a well-known traditional Chinese medicinal herb that has been used for more than 2000 years. In the Chinese Pharmacopoeia, Chaihu is recorded as the dried roots of *Bupleurum chinense* DC and *B. scorzonerifolium* Wild (Yang et al., 2017). In traditional Chinese medicine (TCM), the main medicinal properties of Chaihu are associated with divergent wind heat, evacuation, and antipyretic activity, as well as with the ShuganJieyu and Shengyang trapping functions. Chaihu is widely used in Japan and Korea to treat colds, fevers, chest pains, malaria, middle gas sag, and irregular menstruation (Pan, 2006). Chaihu is also the main ingredient in several established prescriptions and proprietaries used in Chinese medicines, including Chai-Ge-Jieji-Tang, Xiao-Chaihu-Tang, Chaihu-Shugan-Shan, and Chaihu-Baihu-Tang (Zhu et al., 2017).

**FIGURE 1:**
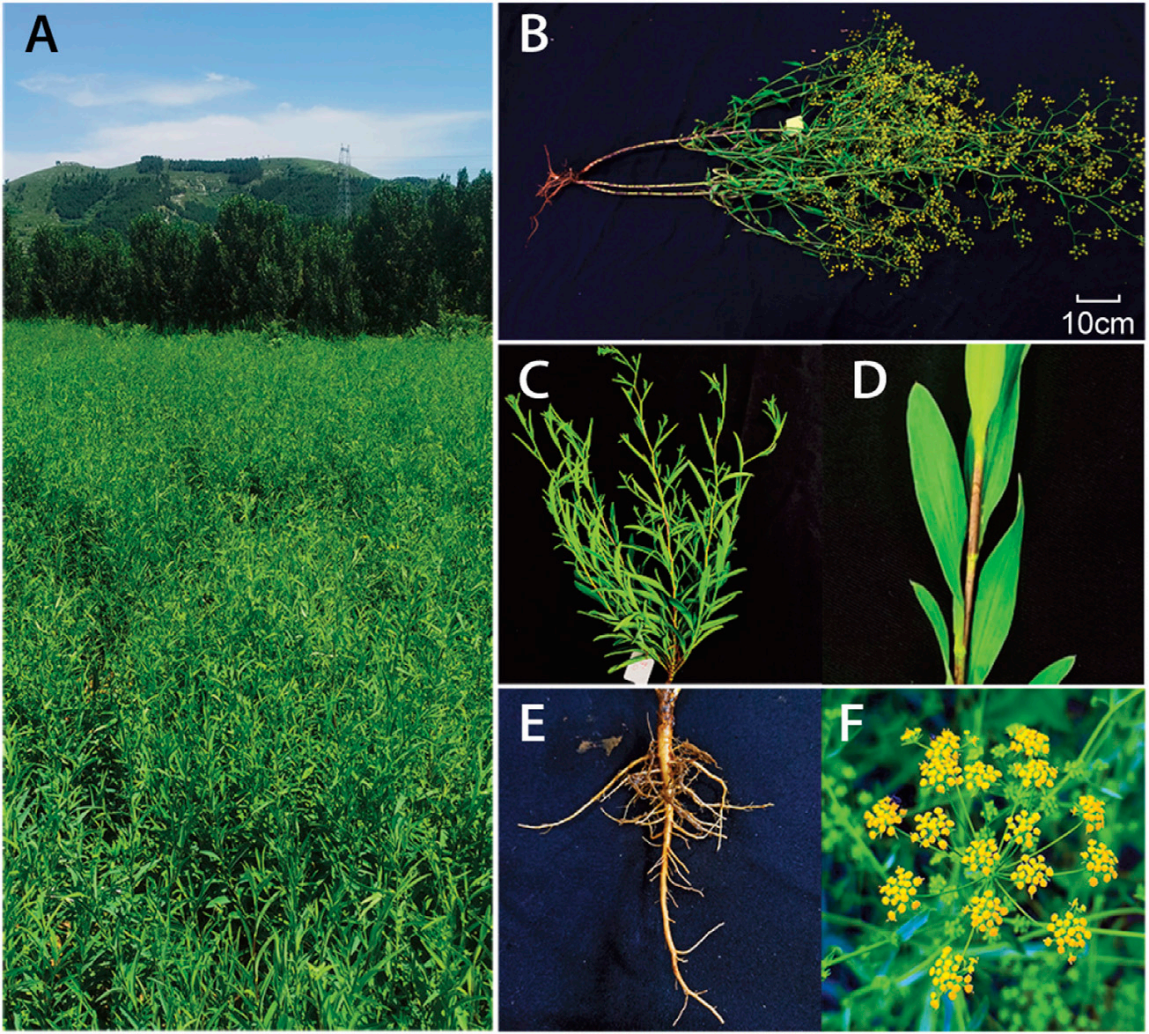
Morphological characteristics of the *B. chinense*. Mature plants in **(A)** and **(B)**, The above-ground plant **(C)** and leaves in the stem **(D)**, root system**(E)**, and flowers **(F)** are shown.

Most *Bupleurum* species are perennial herbs that measure approximately 150 cm in height and contain compound umbels (The flowers are bisexual, possess five stamens, and are usually yellow (more rarely purple)). The fruits are like cremocarps, whereas the leaves are simple, long, slender, and alternate through the entire margin (Figure 1). The genus *Bupleurum* includes 180–190 species that are widely distributed in the northern hemisphere, and they are commonly used in medicinal treatments in Eurasia and North Africa (Ashour and Wink, 2011). *Bupleurum Chinese* DC was imported to China as a processed material and is now predominately grown in rural locations (Lin et al., 2008). The main active components in *Bupleuri radix* are saikosaponins (SSs), and specifically triterpene saponins. More than 120 glycosylated oleanane-types and ursane-types of saponins have been isolated from *Bupleurum*. The most predominant SS monomers in *Bupleurum* are SS-a, SS-c, and SS-d (Xu et al., 2019). These different SSs monomers exhibit different pharmacological effects. Specifically, SS-a and SS-d, isolated from *B. falcatum*, have anti-inflammatory activities (Park et al., 2002). *Bupleurum chinense* DC and its active components (saikosaponins) exhibited immunomodulatory (Yen et al., 1995; Benito et al., 1998), anticancer (Law et al., 2014), antiviral (Cheng et al., 2006), antipyretic (Kang et al., 2008), and hepatoprotective (Yan and Rong, 2011; Teschke, 2014; Dang et al., 2019) effects. The important medicinal role of saikosaponins has led to extensive studies on its biosynthesis in different *Bupleurum* species. A lot of medicinal plant genomes have been released for address of major scientific mysteries at the genome scale (Chen et al., 2018). However, the current lack of genomic information on these species hampers further research on this mechanism.

The synthesis of saikosaponins involves different metabolic steps. In higher plants, triterpenes are synthesized via the mevalonate-dependent isoprenoid pathway, and oxidosqualene is a common precursor in the biosynthesis of triterpenoids (Abe, 2007). The putative SS biosynthetic pathway initiates the isoprenoid pathway, mediates the cyclization of oxidosqualene, and subsequently undergoes oxidation modifications, glycosylation, and other secondary transformations, leading to the formation of various saikosaponin monomers (Trapnell et al., 2012; Lin et al., 2013). In higher plants, the cyclization of oxidosqualene to *β*-amyrin and cycloartenol is catalyzed by *β*-amyrinsynthase (BAS), whereas the cyclization of oxidosqualene to cycloartenol is catalyzed by cycloartenol synthase (CAS) (Suzuki et al., 2002). The BAS enzyme catalyzes the first step in the biosynthesis of saikosaponins in *Bupleurum* (Suzuki et al., 2005). Interestingly, the expression of the transcript encoding BAS corresponding to a group of cytochrome P450 hydroxylases and several UDP-glucuronosyltransferases (UGTs) was increased after the application of MeJA in adventitious root cultures of *B. kaoi*, which correlates with increased saikosaponin production (Chen et al., 2007). Furthermore, some *B. chinense* DC P450s and UGTs exhibit tissue-specific responses to methyl jasmonate (MeJA), and a putative saikosaponin biosynthetic pathway has been derived from the finding of studies on *B. chinense* DC (Sui et al., 2011), *B. falcatum* (Kim et al., 2011) and *B. kaoi* (Chen et al., 2007). A few genes of the enzymes of SSs participating in the biosynthesis of SSs were isolated (Yu et al., 2020). Manipulation of genes involved in saikosaponin biosynthesis, especially those of the BAS, P450, and UGT families, may improve saikosaponin production.

*Bupleurum* species are officially listed in the Chinese and Japanese Pharmacopoeias and in the WHO monographs of commonly used medicinal plants in China and Korea. The important medicinal role of *Bupleurum* species has paved the way for intensive research into the biosynthesis of saikosaponins. However, the available genomic information for *Bupleurum* species remains limited, hindering its utilization. Here, we address this important gap and report a chromosome-level genome assembly B. Chinese DC by integration of Illumina short read sequencing, PacBio single-molecule sequencing and Hi-C sequencing. The novel genome assembly is highly complete and provides an excellent resource for future genomic, biological, and ecological studies on this species. In addition, this genome will facilitate future breeding to imporve phenotypes with higher yields, rapid growth, and disease resistance.

## 2 Materials and Methods

### 2.1 Samples and DNA Sequencing

*Bupleurum chinense* DC specimens were collected from Jinan City, ShanDong Province, China. The genomic DNA was extracted using the TIANamp plant DNA Kit following the manufacturer’s instructions (Tiangen, Beijing, China). The DNA was then sheared using a sonication device to enable the construction of short-insert paired-end (PE) libraries. These short-insert libraries, of size 500 bp, were constructed according to the instructions described in the Illumina Nextera DNA Library Prep Kit (Illumina, United States). All libraries were sequenced on an Illumina X-TEN platform (San Diego, CA, United States). The raw reads were subsequently trimmed for quality using Trimmomatic (v.0.35) software (Bolger et al., 2014). The Illumina sequence adaptors were removed, the low-quality bases at the start and the end of the raw reads were trimmed, and the reads were scanned using a four bp sliding window to further trim them when the average quality per base was lower than 15. The resulting clean data were used for the downstream analysis. For the construction of the PacBio library, we used the genomic DNA of *B. chinense* DC, sheared it to ∼20 kb fragments, and those smaller than 7 kb were filtered out using BluePippin (Sage Science, MA, United States). The filtered DNA was then converted into the proprietary SMRTbell library using the PacBio DNA Template Preparation Kit (Pacbio, United States) following the manufacturer’s instructions. Single Molecule Real Time (SMRT) sequencing was conducted on a PacBio Sequel II sequencing platform using HiFi Bundle (v2) sequencing reagent and the SMRT Cell (8M). The leaves of *B. chinense* DC were fixed in 1% (vol/vol) formaldehyde and then used to prepare *in situ* Hi-C libraries. Nucleus extraction, permeabilization, chromatin digestion, and proximity-ligation treatments were performed as previously described (Xie et al., 2015). MboI (NEB, United States) was used as the restriction enzyme. The libraries were sequenced on an Illumina X-TEN platform with 2 × 150 bp reads. To facilitate the prediction of protein-coding genes, RNA was extracted from the root, stem, leaf, flower, and seed tissues using the TRNzol universal Reagent (TIANGEN Biotech, China). RNA-Seq libraries were prepared using the NEBNext Ultra RNA Library Prep Kit (Illumina, United States), following the manufacturer’s instructions, and sequenced on an Illumina X-TEN platform (San Diego, CA, United States). Quality control of the resulting raw reads was performed using Trimmomatic software.

### 2.2 Genome Size and Heterozygosity Estimation

The sizes of horticultural plants genomes vary greatly (Chen et al., 2019). The distribution of k-mer frequency, also known as the k-mer spectrum, is widely used to estimate genome size (Liu et al., 2020). Accordingly, we used Jellyfish software (v1.1.11) (Marçais and Kingsford, 2011) to estimate the size of the genome with high-quality reads above Q20 from the short-insert size libraries (500 bp). We obtained k-mer (k = 17) ( depth distribution from the Jellyfish analysis and roughly estimated the genome size by dividing the total number of k-mers by their respective coverage. A more accurate estimation of the genome size and heterozygosity were obtained using the software GenomeScope (v1.0.0) (Vurture et al., 2017).

### 2.3 Analysis of Genome Size by Flow Cytometry

Flow cytometry (FCM) assays have been used to measure genome size by comparing the fluorescent intensities of samples with reference standards (Doležel and Bartoš, 2005; Doležel et al., 2007). We used a FACS Calibur (Becton Dickinson, United States) cell sorting system to compare the genomic size of *B. chinense* DC with that of tomato. Plant cells were isolated from 20 mg of fresh tissue using the MGb buffer (45 mM MgCl_2_⋅6H_2_O, 20 mM MOPS, 30 mM Na-Citrate, 1% PVP40, 0.2% Triton x-100, 10 mM Na_2_EDTA, 20 μL/ml β-mercaptoethanol, pH 7.5). Thereafter, we added 50 mg/ml propidium iodide (PI) as the DNA fluorochrome and 50 mg/ml RNase to the isolation buffer. The fluorescence intensities of the stained nuclei of the three samples were measured by FCM. The genomic size of *B. chinense* DC was then estimated based on the relative fluorescence intensities of the different species analyzed.

### 2.4 Karyotype Analysis

The roots of *B. chinense* DC that contained active root apical meristems were obtained from 7-day-old tissue cultures. At the time of collection, the roots measured 1.5–2.0 cm in length. To obtain a large number of cells that were in the metaphase stage, we induced cell mitosis by exposing the cultures to nitrous oxide for 2 h under 1 MPa. The treated root tips were diced and digested to expose the cells to a mixture of 1% pectolyase Y23 and 2% cellulase Onozuka R-10 (Yakult Pharmaceutical, Japan) for 1 h, at 37°*C*. The cells were then collected by centrifugation and re-suspended in 90% acetic acid. The droplets from the cell suspension were then placed on glass slides contained in a box lined with wet paper. The fluorescence staining of the chromosomes was performed using 4′,6-diamidino-2-phenylindole (DAPI), as previously described (Kolano et al., 2013). After DAPI staining, the dispersed metaphase chromosome cells were counted under a fluorescence microscope (Zeiss LSM880, Germany). Accurate karyotyping was confirmed by fluorescence *in situ* hybridization (FISH), as previously described (Jiang et al., 2019). A telomere-specific-repeat probe (5′-TTTAGGGTT TAGGGTTTAGGG-3′) was used to confirm the number of intact chromosomes, and a 18s rDNA repeat sequence probe was used to identify multiple copies of chromosomes.

### 2.5 Genome Assembly and Quality Control

We integrated the assembled PacBio HiFi reads and paired-end Hi-C reads using HiFiasm software (v0.14) with the parameters -t 32 and -f 39 (Cheng et al., 2021). Illumina short reads were then aligned to the corrected HiFiasm contigs using BWA-MEM (v0.7.17; Li and Durbin (2009)), and Pilon (v1.2; (Walker et al., 2014)) was used to correct errors in the contigs. The Hi-C data were independently analyzed in the HiC-Pro pipeline (default parameters and LIGATION_SITE = GATC; (Servant et al., 2015)). The 3D-DNA pipeline was used to assign the order and orientation of each group (Dudchenko et al., 2017), and the contact maps were plotted using HiCPlotter (Akdemir and Chin, 2015). To evaluate the completeness of the genome assembly, we used conserved core genes to run BUSCO software on the *B. chinense* DC genome assembly.

### 2.6 Genome Annotation

RepeatModeler were used to build a *de novo* library on the basis of our genome sequences, and then, by using the build library as database, RepeatMasker was utilized to classify the types of repetitive sequences. On the other hand, TEs in DNA and protein levels were identified by aligning genome sequences against the Repbase TE library. To ensure the integrity of the genes in the subsequent analyses, we masked all repeat sequences (except low complexity and simple repeats) from this analysis. The identification of protein-coding regions and the subsequent gene predictions was performed using a combination of *ab initio*, homology-based, and transcriptome-based prediction methods. The *ab initio* coding gene prediction was conducted using Augustus (v2.5.5; (Stanke et al., 2008)) and GeneMark software (v4.32; (Besemer et al., 2001)). For the homology-based prediction, we downloaded homologous protein sequences in *Apium graveolens* (GCA_009905375.1) and *Daucus carota* (GCF_001625215.1) from the NCBI database and aligned them to our newly assembled genome. Subsequently, we used Exonerate software (v2.2.0; (Slater and Birney, 2005)) to build gene structures based on the homologous alignments. For transcriptome-based predictions, we mapped the RNA-Seq data against the assembly using Tophat (v2.1.0; (Trapnell et al., 2009)). We then used Cufflinks (v2.2.1; Trapnell et al. (2012)) on the transcripts resulting from the Tophat analysis to perform gene model analysis. We then integrated the results from the three prediction methods using EvidenceModeler (EVM) and obtained a non-redundant gene set (Haas et al., 2008). Functional annotations were conducted on the obtained gene set using BLASTP with an E-value of 1e-05 against the NCBI-NR, SwissProt, and KOG databases. Protein domains were mapped against the InterPro and Pfam databases using InterProScan and HMMER (Finn et al., 2011). The putative pathways to which the different genes belong were derived from genes mapped against the KEGG database (Kanehisa and Goto, 2000). The Gene Ontology (GO) terms for the different genes were extracted from the corresponding InterProscan or Pfam results.

### 2.7 Haplotype Comparison

We performed all-against-all whole-genome alignments of the two haplotype’s genomes using nucmer in MUMmer (v4.0.0; (Marçais et al., 2018)) allowing for multiple matches (–maxmatch option). We filtered the alignments for a minimal identity of 90% and a minimal single match length 100bp using the delta-filter in MUMmer. We then extracted the alignment coordinates using show-coords. Finally, structural variants were identified using the SyRi (v1.3 (Goel et al., 2019). Gene synteny between haplotype 0 and 1 assemblies was analyzed using MCscanX (Wang et al., 2012). GeneWise (Birney and Durbin, 2000; Birney et al., 2004) was used to align the unique genes in haplotype 0 against the genome sequence of haplotype 1 to determine homologous sequences.

### 2.8 Phylogenetic Analysis and Divergence Time Estimation

Phylogenetic analysis was performed by constructing phylogenetic trees based on single copy genes in orthogroups within 17 species (Supplementary Table S5). We performed clustering analyses on the protein sequences using the Markov clustering program OrthoFinder (v2.5.3; (Emms and Kelly, 2015)). The peptide sequences were also searched against the NCBI-NR database using an all-versus-all BLASTP approach, with a threshold value of E-value ≤ 1e-05 ≤ . The sequences were then clustered by MCL with an inflation value of 1.5. Orthologous alignments were produced using MUSCLE (v3.6; (Edgar, 2004)), and then concatenated into a unique multiple sequence alignment using an in-house-developed Perl script. A neighbor-joining phylogenetic tree was reconstructed using MEGA5 software. We estimated the molecular clock and the divergence times using a combined analysis with the programs r8s (Sanderson, 2003) and RAxML (v8.2.10; (Stamatakis, 2014)). Maximum likelihood phylogeny and respective branch lengths were calculated with RaxML using 1,000 bootstrap replicates. The fossil-derived timescale and the evolutionary history of these species were obtained from TIMETREE (Kumar et al., 2017). We were not able to obtain confidence intervals for the r8s analysis due to a lack of convergence between the subsamples.

### 2.9 Expansion and Contraction of the Gene Families

To understand the evolutionary relationship between *B. chinense* DC and other plant species, we performed a systematic comparison, including different genes. Specifically, we used the full protein-coding genes from haplotype 0 of *B. chinense* DC and other 16 species (Supplementary Table S5). As the gain and loss of genes are the primary contributors to functional changes in different species, we sought to obtain a better understanding of the evolutionary dynamics of gene gain/loss by determining the expansion and contraction of the orthologous gene clusters among these 17 species. We used CAFE software (v4.0; (De Bie et al., 2006)) to perform computational analysis of the evolution of the gene families, and then defined the expansion and contraction of the gene families by comparing the differences in cluster sizes between each of the current species and their respective ancestors. We used the random birth and death process model in CAFE to identify gene gain and loss events along each branch of the RAxML tree. We then compared the expanded and contracted gene families (in comparison to ancestors) identified in the different species with those of *B. chinense* DC, to understand the evolution of the gene families in the latter.

### 2.10 Identification and Analysis of Genes Involved in the Biosynthesis of Saikosaponins

The sequences of genes associated with the biosynthesis of saikosaponins were downloaded from UniProt (https://www.uniprot.org) and placed in a new database. This included genes related to the early steps in the saikosaponin biosynthetic pathway, namely oxidosqualene cyclase (OSC) genes and UDP-Glycosyltransferase genes. These genes were identified using BLASTP against the database with an E-value cutoff of 1e-10. The genes that were consistently annotated with the NCBI-NR, Uniprot, and KEGG databases were used to classify the genes associated with saikosaponin biosynthesis.

### 2.11 Identification and Analysis of the Cytochrome P450 Family and Subfamily

All protein sequences identified in the *B. chinense* DC genome were compared with those in the Pfam database. Among these, those containing a P450 domain were classified as candidate P450 genes. The sequences of the P450 genes obtained from http://cyped.uni-stuttgart.de were then used as BLASTP input sequences, with an E-value ≤ 1 e-07 in comparison to the gene family and subfamily. All P450 protein sequences were aligned using MUSCLE software, after which a neighbor-joining phylogenetic tree was constructed using MEGA5.

## 3 Results

### 3.1 Genome Size Estimation of *B. chinense* DC

The genome size of *B. chinense* DC was estimated by flow cytometry before genome sequencing, where the tomato genome (comprising 900 Mb) was used as a reference. The relative fluorescence intensity of *B. chinense* DC nuclei indicated that its genome size is approximately 62% smaller than that of tomato, which is approximately 540 Mb. Paired-end sequencing library data and k-mer frequency analysis were then used to estimate the genome size of *B. chinense* DC as 623.15 Mb, a similar albeit higher estimation than that when the flow cytometry data were used (Supplementary Figure S1). The genome-wide heterozygosity rate was estimated as 1.94%.

### 3.2 Karyotype of *B. chinense* DC

**FIGURE 2:**
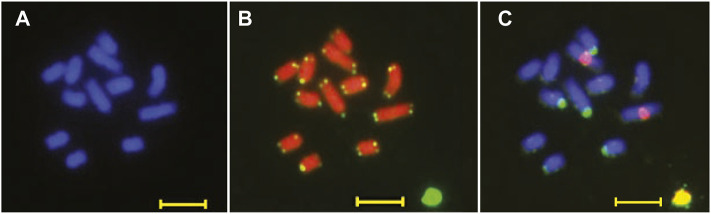
Karyotype pictures of B. chinense DC using **(A)** DAPI staining, **(B)** fluorescence *in situ* hybridization (FISH) with a telomere specific repeat probe, and **(C)** an 18 s rDNA repeat sequence probe. Bar = 5 μm.

We analyzed the karyotype of *B. chinense* DC to determine the chromosome number and ploidy. We observed and recorded more than 20 root cell chromosome samples using a high-resolution metaphase chromosome preparation technology. We estimated that the *B. chinense* DC genome is composed of 12 chromosomes, by DAPI staining, with a size range between 0.5 and 1.5 μm (Figure 2A). The number of chromosomes was confirmed by fluorescence *in situ* hybridization (FISH) and telomere repeat probes, which indicated a clear fluorescence signal from each of the telomeres at the end of the different chromosomes (Figure 2B). We then analyzed the ploidy using FISH with 18S rDNA repeat sequence probes. The 18S rDNA hybridization signal showed that all cells in the sample contained two pairs of chromosomes (Figure 2C), indicating that the species is diploid, with 2n = 12.

### 3.3 *De novo* Genome Assembly of *B. chinense* DC

**FIGURE 3:**
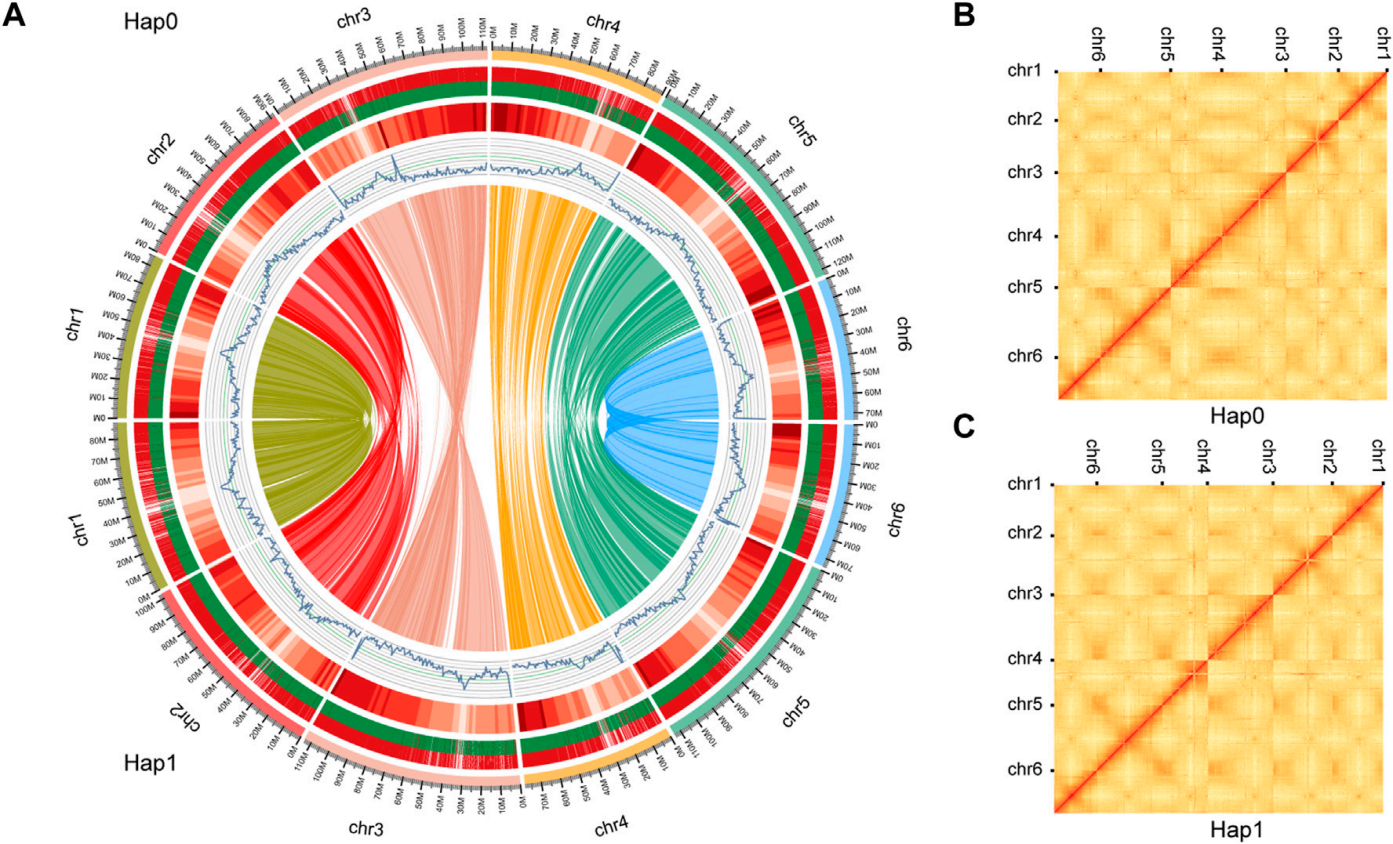
Schematic representation of the genomic characteristics and Hi-C contact map of haplotypes 0 and 1. **(A)** Schematic representation of the genomic characteristics of haplotypes 0 and 1. From the outer-ring: Track 1: the 6 pseudo chromosomes comprising haplotypes 0 and 1. genome; Track 2: Protein-coding genes present in each of the chromosomes. Long and short bars represent genes on the forward and reverse strands, respectively;Track 3: The distribution of the gene density using 1-Mb sliding windows. Higher densities are shown in darker red. Track 4: The distribution of the GC content. Track 5: The distribution of the repeat density using 1-Mb sliding windows. Track 6: Gene synteny between haplotype 0 and 1 assemblies was analyzed using MCscanX. The Hi-C contact map of the haplotype 0 **(B)** and haplotype 1 **(C)** genome. The color bar shows the contact density from white (low) to red (high).

We obtained 28.3 Gb of clean sequencing data from the Illumina platform, 417.4 Gb from the PacBio Sequel platform, and 81.3 Gb from the Hi-C library (Supplementary Table S1). This allowed us to obtain a high-quality haplotype-resolved chromosome-level genome assembly. Approximately 92.90 and 95.06% of sequences were anchored onto pseudochromosomes in the haplotype 0 and haplotype 1, respectively. The genome size of the final assembly for haplotype 0 was 621.42 Mb with contig N50 of 16.86 Mb. The genome size of haplotype 1 was 600.48 Mb with contig N50 of 23.90 Mb (Table 1, Supplementary Table S3, Figure 3). SNP calling was performed to evaluate sequence variations between haplotypes 0 and 1 using MUMmer with the parameter -maxmatch. To assess the accuracy and completeness of the *B. chinense* DC genome assembly, we then used BUSCO (v3.0.2; (Waterhouse et al., 2018)) to evaluate the integrity of the genome. Haplotype 0 showed over 96.7% coverage of the viridiplantae orthologous gene set, and haplotype 1 showed over 96.2% coverage (Supplementary Table S4, Supplementary Figure S2). These results indicated that the two haplotype assemblies covered most of the coding regions, which further confirmed the quality of the genome assembly.

**TABLE 1:** Genome assembly and annotation statistics for *B. chinense* DC.

Haplotypes	Hap0	Hap1
Length of the scaffolds (bp)	621,267,366	600,371,836
Length of the contigs (bp)	621,420,202	600,482,800
Number of scaffolds	2,912	1,775
Number of contigs	1,382	664
Scaffolds N50 (bp)	92,254,985	102,676,127
Contigs N50 (bp)	16,864,651	23,895,762
Scaffolds N90 (bp)	73,672,175	74,133,959
Contigs N90 (bp)	39,561	85,203
GC Content (%)	33.5	33.52
N Content (%)	0.024	0.018
Gene number	45,909	35,805
Gene density (gene_number/100 kb)	7.39	5.96
Gene average length (bp)	1,145.8	1,211.9
Exon number per gene	4.78	5.30
Intron number per gene	3.78	4.30
Exon average length (bp)	239.6	228.5
Intron average length (bp)	535.6	583.2
Genome GC (%)	33.51	33.53
Exon GC (%)	42.10	42.03
Intergenic region average length (bp)	7,288.0	9,055.0

### 3.4 Genome Annotation

Our analysis showed that the repeat elements identified in the haplotype 0 genome constituted 53.94% of the whole genome, including 0.47% of the satellite sequences and 53.47% of the interspersed repeats. Among the latter, the long terminal repeats (LTR) elements comprised 25.93% of the genome, whereas DNA elements represented only 4.10% (Supplementary Table S2, Supplementary Figure S3). Haplotype 1 genome constituted 55.16% of the whole genome (Supplementary Table S2). We then employed *de novo* methods using transcriptome data and homolog-based approaches to predict the gene models. These predictions were integrated by EVM into a weighted and non-redundant gene structure consensus model. A total of 45,909 protein-coding genes were identified in haplotype 0 with an average CDS length of 1,145.80 bp, whereas 35,805 protein-coding genes were identified in haplotype 1 with an average CDS length of 1,211.90 bp. (Table 1; Figure 3). Genes from Haplotype 0 were used in the following phylogenetic and saikosaponin biosynthesis analysis. The gene sequences were subsequently aligned with those in several public databases [NCBI-NR, GO (Supplementary Figure S4), Swiss, KOG (Supplementary Figure S5), and KEGG] to obtain functional annotations. We were able to map a total of 45,770 genes (99.70%) to at least one database, with 22,124 genes annotated in all four databases (Supplementary Figure S6).

### 3.5 Haplotype Comparison

SNP calling was performed to evaluate sequence variation between haplotypes 0 and 1 using MUMmer with all default settings. Heterozygosity between the haplotypes was found to be 1.89%, which was consistent with the k-mer analysis. Structure variations (SVs) were identified between the two haplotypes, including 537 syntenic regions, 1,023 translocations, 121 inversions, 390 and 451 duplications in haplotypes 0 and 1, respectively. The collinearity relationship between the two haplotypes was analyzed, and the synteny analysis revealed that 927 collinear blocks were identified between the two haplotypes. Overall, 51,398 collinearity genes were identified, accounting for 62.69% of all genes. Shared genes and unique genes between the two haplotypes were identified, and a total of 70,801 shared genes were identified, accounting for 86.64% of all genes in the two genomes. Additionally, 8,362 unique genes were identified in haplotype 0, accounting for 18.21% of all haplotype 0 genes, and 2,551 unique genes were identified in haplotype 1, accounting for 7.12% of all genes in the haplotype 1 genome. The comparative genomic analysis implies that the difference in the number of genes between the two haplotypes due to unique genes. In order to verify that difference in the number of genes between the two haplotypes was not caused by gene model prediction, we compared the unique genes in haplotype 0 with the genome of haplotype 1. We compared the unique genes in haplotype 0 with the genome of haplotype 1 using Genewise, less than 4.53% of unique genes in haplotype 0 have homologous sequences in haplotype 1. These findings show that the observed difference in the number of genes between the two haplotypes is likely due to natural inherent differences between the two haplotypes’ genomes.

### 3.6 Phylogenetic and Divergence Analyses

**FIGURE 4:**
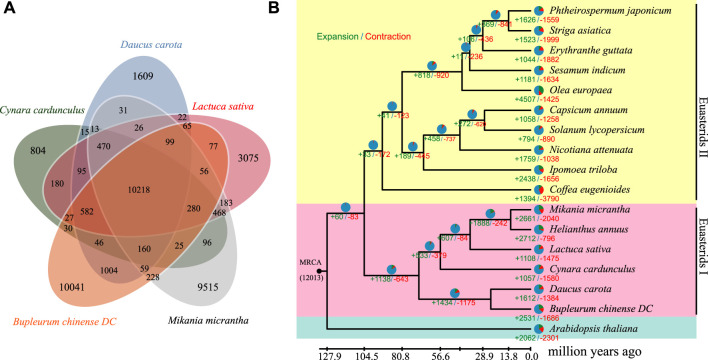
Genome evolution analysis of *B. chinense* DC. **(A)** Venn diagram of the orthologous genes shared between *Lactuca sativa*, *Cynara cardunculus*, *Mikania micrantha*, *Daucus carota*, and *Bupleurum chinense* DC. **(B)** The phylogenetic relationship between *B. chinense* DC and 16 other species. The number of gene gains (+) and losses (-) is shown on each branch, and are also displayed as pie charts:green, red, and blue corresponding to gene gain, loss, and neither gain nor loss, respectively. The divergence times between the different species are displayed below the tree.

To examine the evolutionary relationship between *B. chinense* DC and other species, we analyzed the protein sequences of 17 different species. A 5-way comparison of four species closely related, *Lactuca sativa*, *Cynara cardunculus*, *Mikania micrantha*, and *Daucus carota*, showed that 10,218 gene families 269 were shared among them, which were suggestive of higher similarity (Figure 4A). A total of 96,612 gene families were identified among these 17 species, of which 7,414 families were present across all species (Supplementary Table S5). Supplementary Table S5A total of 252 single-copy orthologous genes were then selected for further phylogenetic analysis. Thereafter, we constructed a maximum likelihood phylogeny using RAxML (Figure 4B). Based on the obtained phylogeny and the fossil record, we were able to date the divergence time between *B. chinense* DC and *D. carota* to ∼57.30 million years ago, and the divergence between the euasterid I and II clades to ∼122.74 million years ago.

### 3.7 Expansion and Contraction of Gene Families in the *B. chinense* DC Genome

To compare different genomic traits across species, we performed a comparative genomic analysis on the 17 species using CAFE software (Supplementary Table S6). We found that 260 and 342 gene families were significantly expanded and contracted in the *B. chinense* DC genome, respectively (*p* < 0.05). Furthermore, we uncovered 29 and 94 significantly enriched KEGG pathways from the expanded and contracted gene families, respectively (FDR cut-off < 0.05). The enrichment analyses suggested that many expanded gene families are involved in biological processes associated with the biosynthesis of isoquinoline alkaloid, tyrosine, and anthocyanin (Supplementary Figure S7).

### 3.8 Saikosaponin Biosynthesis

**FIGURE 5:**
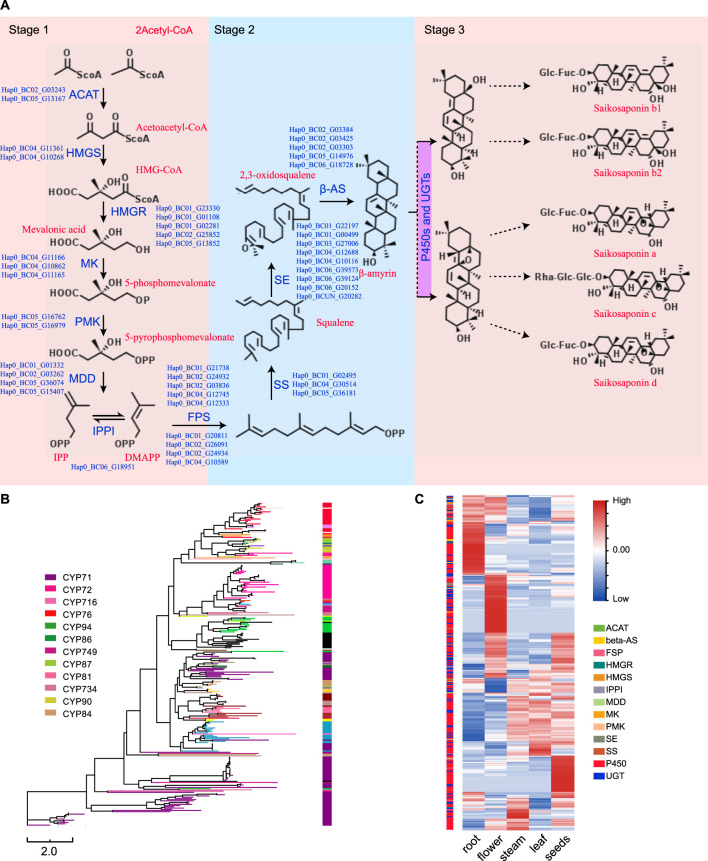
Identification and analysis of the genes involved in the biosynthesis of saikosaponins. **(A)** The SS biosynthetic pathway. **(B)** A neighbor-joining phylogenetic tree including the P450 protein sequences. Each color represents a P450 family or subfamily. **(C)** Heat map showing the expression levels of the P450s, UGTs, and other genes involved in the biosynthesis of saikosaponins based on the RNA-Seq data from the root, stem, leaf, flowers, and seeds.

Saikosaponins are one of the main active components in *B. chinense* DC. In total, there are more than 120 glycosylated oleanane-type and ursane-type saponins that have been isolated from different *Bupleurum* species (Lin et al., 2013; Sui et al., 2011). Specifically, the SS biosynthetic pathway can be divided into three different stages (Figure 5A): the first stage occurs during the formation of isopentenyl pyrophosphate (IPP) and dimethylallyl pyrophosphate (DMAPP); the second stage corresponds to the formation of the triterpene skeleton (b-amyrin); and the third stage occurs during and after the modification of this skeleton (Sui et al., 2011). In this study, we were able to identify the different genes associated with the first and second stages of saponin biosynthesis, and determined their genome-wide copy numbers. Furthermore, we identified 80 UGT and 266 P450 genes in the *B. chinense* DC genome, as well as 266 CYP sequences that were classified into 29 families, according to standard CYP nomenclature (Table 2 and Figure 5B). Based on current knowledge, no definite sequence features could be used to identify the specific P450s and UGTs involved in the modifications of the triterpene skeleton. To identify the P450 and UGT genes involved in SS biosynthetic pathways, we investigated their levels of expression using RNA-Seq data from different tissues, including the root, stem, leaf, flowers, and seeds. Our analysis showed that 46 P450 genes were specifically highly expressed in the roots, and that their expression profiles were highly correlated with that of the BAS gene (correlation coefficient *r* > 0.9; Figure 5C). Additionally, we found 9 UGT genes that were specifically highly expressed in the roots, and whose expression profiles were highly correlated with the BAS gene (correlation coefficient *r* > 0.9); Figure 5C). This suggests that some of these P450 and UGT genes might be involved in the biosynthesis of saikosaponins. In this study, the candidate P450 and UGT genes in the third stage of SS biosynthetic were determined for the first time, which provided a genetic basis for SS biosynthetic *in vitro*.

**TABLE 2:** Genes involved in the biosynthesis of saikosaponins and their respective copy numbers in the *B. chinense* DC genome and *D. carota* genome.

Stage	Gene	*B. chinense*	*D. carota*
Stage 1	ACAT	2	3
	HMGC	2	1
	HMGR	5	5
	MK	3	1
	PMK	2	3
	MDD	4	1
	IPPI	1	1
Stage 2	FPS	9	9
	SS	3	3
	SE	9	7
	*β*-AS	5	4
Stage 3	P450s	266	207
	UGTs	80	190

## 4 Conclusion

In this study, we assembled a high-quality haplotype-resolved chromosome-level genome for *B. chinense* DC using a combined strategy encompassing three distinct sequencing technologies: Illumina short reads, PacBio single-molecule, and Hi-C. The assembled genome contains two haplotypes, haplotype 0 (6 pseudo-chromosomes, 2,912 contigs with an N50 length of 16.86 Mb) and haplotype 1 (6 pseudo-chromosomes, 1,775 contigs with an N50 length of 23.90 Mb). Haplotypes 0 and 1 contain 96.7 and 96.2% of the core genes, as shown by BUSCO analysis, respectively. We presented different genomic features and performed phylogenetic and gene family evolution analyses in *B. chinense* DC plants. We implemented functional enrichment analyses that suggested that the expanded gene families in the *B. chinense* DC genome are associated with the biosynthesis of anthocyanins, sesquiterpenoids, and triterpenoids. Finally, we identified and analyzed the genes involved in the biosynthesis of saikosaponins, and in particular, P450s and UGTs, which provide important insights into this important metabolic process. The newly assembled *B. chinense* DC genome constitutes an excellent resource for genomic, biological, and ecological studies of this species, and will facilitate future molecular breeding for high yield, rapid growth, and disease resistant phenotypes.

## Data Availability

The datasets presented in this study can be found in online repositories. The names of the repository/repositories and accession number(s) can be found in the article/Supplementary Material.
