# Supplementary material for: Chromosome-Level Genome Assembly of Bupleurum chinense DC Provides Insights Into the Saikosaponin Biosynthesis
Source: Front Genet. 2022 Mar 31;13:878431. doi: 10.3389/fgene.2022.878431 (PMC9008701; doi:10.3389/fgene.2022.878431)
Supplement: Supplementary file 1 [file DataSheet1.pdf]

Supplementary Material

1 SUPPLEMENTARY TABLES AND FIGURES

1.1 Tables

Table S1. Summary statistics for the clean sequencing data obtained from *B. chinense* DC via Illumina and PacBio sequencing

Clean Reads	Illumina Sequencing	PacBio Sequencing	HiC Sequencing
Size of library	500 bp	20 kb	500 kb
Number of Reads	96,217,543 *2	37,439,559	234,005,013 * 2
Average length of Reads	147 bp	11,149 bp	149 bp
Total Bases	28,308,402,172 bp	417,402,430,850 bp	69,676,216,418 bp
Sequencing Depth	∼45×	∼400×	∼112×

Table S2. Summary statistics for the repeat elements found in the B. chinense DC genome assembly using both the RepeatModeler and RepeatMasker software

Biological classification	Number of elements*		Length occupied (bp)		Percentage of sequence (%)	
Sample id	Hap0	Hap1	Hap0	Hap1	Hap0	Hap1
Retroelements	164,161	161,197	171,956,385	171,509,491	27.67	28.56
SINEs:	1,375	1,304	176,035	179,437	0.03	0.03
Penelope:	4,263	4,143	405,200	386,388	0.07	0.06
LINEs:	27,488	26,525	10,635,245	9,930,551	1.71	1.65
CRE/SLACS	2	1	99	77	0.00	0.00
L2/CR1/Rex	7,435	7,275	688,127	650,945	0.11	0.11
R1/LOA/Jockey	945	833	60,831	53,660	0.01	0.01
R2/R4/NeSL	991	951	58,008	65,854	0.01	0.01
RTE/Bov-B	627	629	262,305	278,878	0.04	0.05
L1/CIN4	11,433	10,952	8,540,537	7,915,019	1.37	1.32
LTR elements:	135,298	133,368	161,145,105	161,399,503	25.93	26.88
BEL/Pao	1,885	1,803	594,557	575,138	0.01	0.01
Ty1/Copia	69,211	68,681	105,093,806	105,842,644	16.91	17.63
Gypsy/DIRS1	53,894	53,013	52,838,668	52,531,499	8.50	8.75
Retroviral	4557	4,384	502,608	482,640	0.08	0.08
DNA transposons	158,600	153,842	25,495,017	24,982,063	4.10	4.16
hobo-Activator	37,611	36,946	6,324,414	6,236,678	1.02	1.04
Tc1-IS630-Pogo	13,667	13,171	260,075	2,479,060	0.42	0.41
En-Spm	0	0	0	0	0.00	0.00
MuDR-IS905	0	0	0	0	0.00	0.00
PiggyBac	1,636	1,602	481,144	459,830	0.08	0.08
Tourist/Harbinger	7,966	7,643	1,575,973	1,483,073	0.25	0.25
Other(Mirage, P-element,Transib)	3,597	3,559	157,855	152,899	0.03	0.03
Rolling-circles	10,155	9,966	2,798,015	2,745,836	0.45	0.46
Unclassified	362,136	351,731	134,825,449	131,502,700	21.70	21.90
Total interspersed repeats:			332,276,851	327,994,254	53.47	54.62
Small RNA:	16	12	1,064	721	0.00	0.00
Satellites:	8,205	8,893	2,928,165	3,245,441	0.47	0.54
Simple repeats:	4,670	4,575	1411390	1417698	0.23	0.24
Low complexity:	25	27	3576	4753	0.00	0.00

Table S3. Number of contigs anchored with Hi-C

Name	Number of contigs		Length (bp)		Number of gene models	
Sample ID	Hap0	Hap1	Hap0	Hap1	Hap0	Hap1
Chromosome01	115	122	85,490,115	88,332,808	5,527	4,464
Chromosome02	307	153	92,254,985	102,676,127	5,812	6,735
Chromosome03	426	294	112,677,120	113,799,518	8,333	7,792
Chromosome04	337	272	90,131,553	78,390,962	6,154	4,574
Chromosome05	294	234	123,098,921	113,474,231	8,793	7,545
Chromosome06	57	42	73,672,175	74,133,959	4,836	4,584

Table S4. Estimation of core genes in the B. chinense DC genome assembly and genesets using the BUSCO software

Sample id	Number in assembly Hap0/Hap1	Percentage in assembly Hap0/Hap1	Number in genesets Hap0/Hap1	Percentage in genesets Hap0/Hap1
Complete BUSCOs	404/400	95.1/94.1	408/402	96.0/94.5
Complete and single-copy BUSCOs	343/344	80.7/80.9	386/378	90.8/88.9
Complete and duplicated BUSCOs	61/56	14.4/13.2	22/24	5.2/5.6
Fragmented BUSCOs	7/9	1.6/2.1	3/5	0.7/1.2
Missing BUSCOs	14/16	3.3/3.8	14/18	3.3/4.3
Total BUSCO groups searched	425/425	100/100	425/425	100/100

Table S5. Summary of the orthologous gene clusters analyzed in the 17 selected species

Species name	GenBank accesion	# coding genes	# gene families	# genes in gene families	# specific gene familes	Avg. # of genes per gene family
Arabidopsis thaliana	GCF_000001735.3	27,446	12,013	21,861	4,178	1.82
Bupleurum chinense DC	-	45,909	13,781	35,318	9,216	2.56
Capsicum annuum	GCF_000710875.1	29624	13,030	27,653	1,849	2.12
Coffea eugenioides	GCF_003713205.1	28,455	12,667	26,373	1,794	2.08
Cynara cardunculus	GCF_001531365.1	26,326	12,825	25,589	684	2.00
Daucus carota	GCF_001625215.1	31,530	13,099	29,971	1,415	2.29
Erythranthe guttata	GCF_000504015.1	26,923	12,826	25,328	1,314	1.97
Helianthus annuus	GCF_002127325.2	54,910	13,580	45,266	8,114	3.33
Ipomoea triloba	GCF_003576645.1	29,727	12,507	27,873	1,622	2.23
Lactuca sativa	GCF_002870075.1	34,755	13,107	31,593	2,816	2.41
Mikania micrantha	GCA_009363875.1	46,351	13,034	36,945	8,893	2.83
Nicotiana attenuata	GCF_001879085.1	33,274	13,242	30,972	2,028	2.34
Olea europaea	GCF_002742605.1	38,604	13,232	34,972	3,262	2.64
Phtheirospermum japonicum	GCA_014905375.1	30,299	13,009	26,294	3,833	2.02
Sesamum indicum	GCF_000512975.1	23,570	12,601	23,133	405	1.84
Solanum lycopersicum	GCF_000188115.3	25,163	12,907	24,521	622	1.90
Striga asiatica	GCA_008636005.1	33,426	12,357	23,161	9,689	1.87

Table S6. Results of the gene family analysis implemented using the CAFE software

Species	Expanded families	Significantly expanded families (P <0.05)	Families with no change	Contracted families	Significantly contracted families (P <0.05)
Arabidopsis thaliana	2,062	239	7,650	2,301	430
Bupleurum chinense DC	2,531	260	7,301	1,686	342
Capsicum annuum	1,058	262	9,154	1,258	193
Coffea eugenioides	1,394	272	6,733	3791	452
Cynara cardunculus	1,057	147	8,918	1,580	435
Daucus carota	1,612	320	8,525	1,384	235
Erythranthe guttata	1,044	219	8,704	1,882	345
Helianthus annuus	2,712	495	7953	796	167
Ipomoea triloba	2,438	332	7598	1,656	308
Lactuca sativa	1,108	245	8948	1,475	315
Mikania micrantha	2,661	244	6760	2,040	418
Nicotiana attenuata	1,759	266	8,801	1,038	275
Olea europaea	4,507	492	5,857	1,425	211
Phtheirospermum japonicum	1,626	378	8,256	1,559	176
Sesamum indicum	1,181	162	8,873	1,634	389
Solanum lycopersicum	794	153	9,786	890	245
Striga asiatica	1,523	141	7,920	1,999	450

Table S7. Summary statistics for the clean sequencing data obtained from *B. chinense* DC

Source tissue	DNA or RNA	Library Type	Sequencing Platforms	Number of Reads	Bases (GB)	Accession ID
leaves	DNA	15 kb	PacBio Sequel	8,920,910	103.53	SRR13364357
leaves	DNA	15 kb	PacBio Sequel II	37,439,559	417.40	SRR16122634
leaves	DNA	500 bp	Illumina X-ten	98,267,480 x 2	29.48	SRR13364359
leaves	DNA	Hi-C	Illumina X-ten	242,298,981 x 2	72.69	SRR13364356
leaves	RNA	300 bp	Illumina X-ten	32,987,411 x 2	9.89	SRR17234912
root	RNA	300 bp	Illumina X-ten	30,626,143 x 2	9.18	SRR17234911
stem	RNA	300 bp	Illumina X-ten	34,738,902 x 2	10.42	SRR17234910
flowers	RNA	300 bp	Illumina X-ten	26,413,066 x 2	7.92	SRR17234909

1.2 Figures

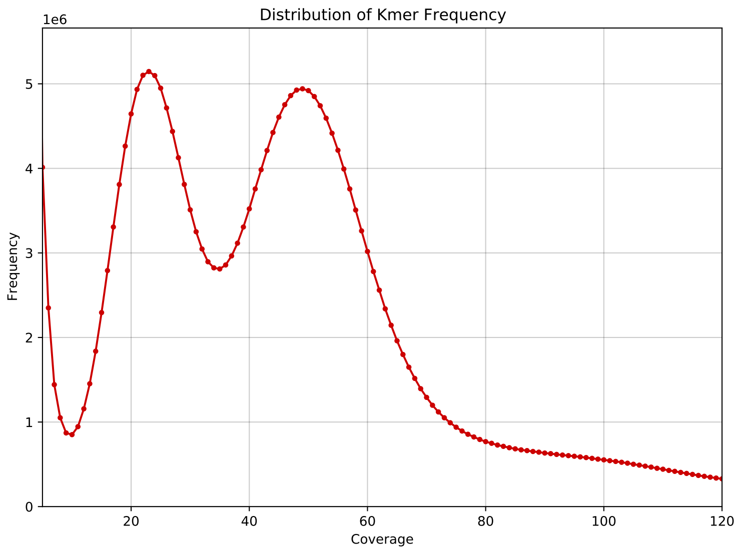

Figure S1. Estimation of the *B. chinense* DC genome size using k-mer analysis.

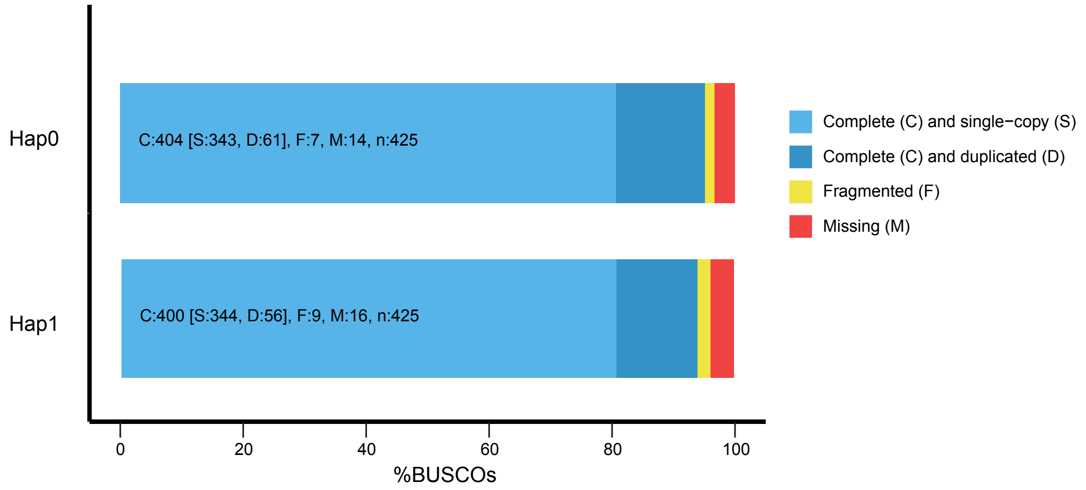

Figure S2. Estimation of the core genes in the *B. chinense* DC genome assembly using the BUSCO software.

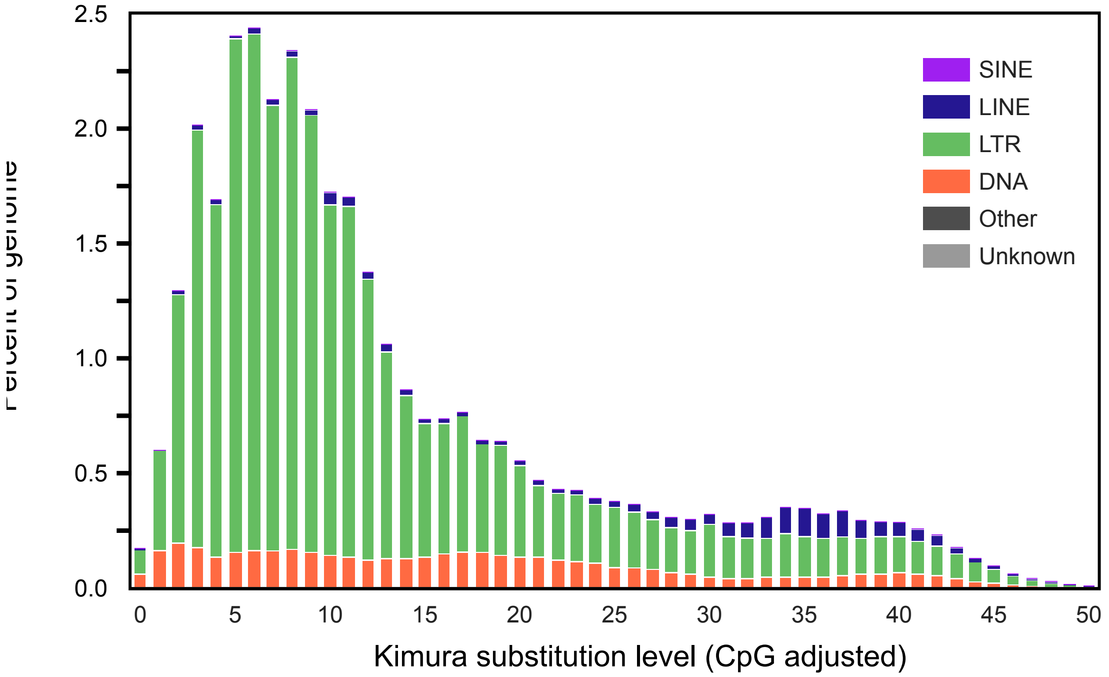

Figure S3. Summary statistics of the repeat elements found in the *B. chinense* DC genome assembly using both the RepeatModeler and RepeatMasker software.

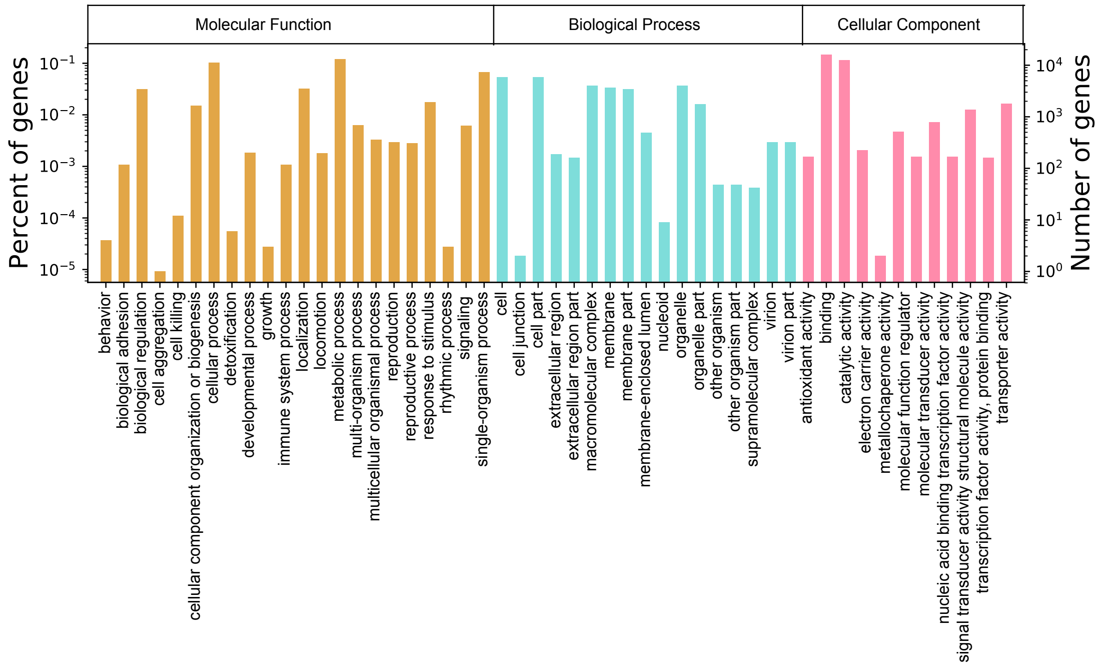

Figure S4. GO analysis and functional classification of the protein coding genes in the *B. chinense* DC genome.

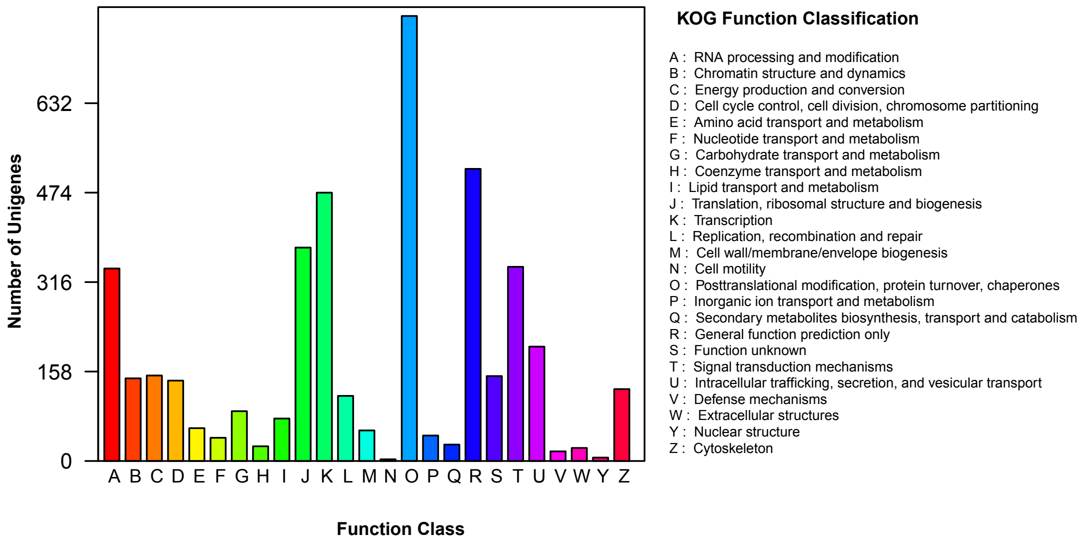

Figure S5. KOG analysis and functional classification of the protein coding genes in the *B. chinense* DC genome.

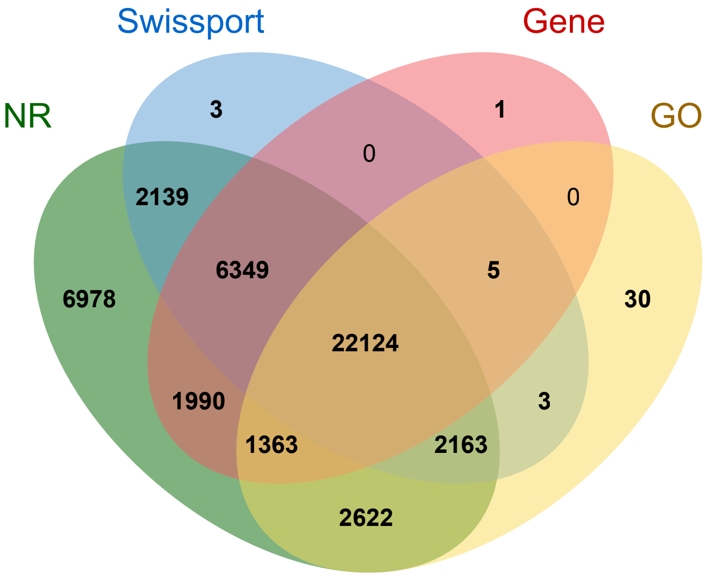

Figure S6. Venn diagram showing the statistics of the functional annotation analysis.

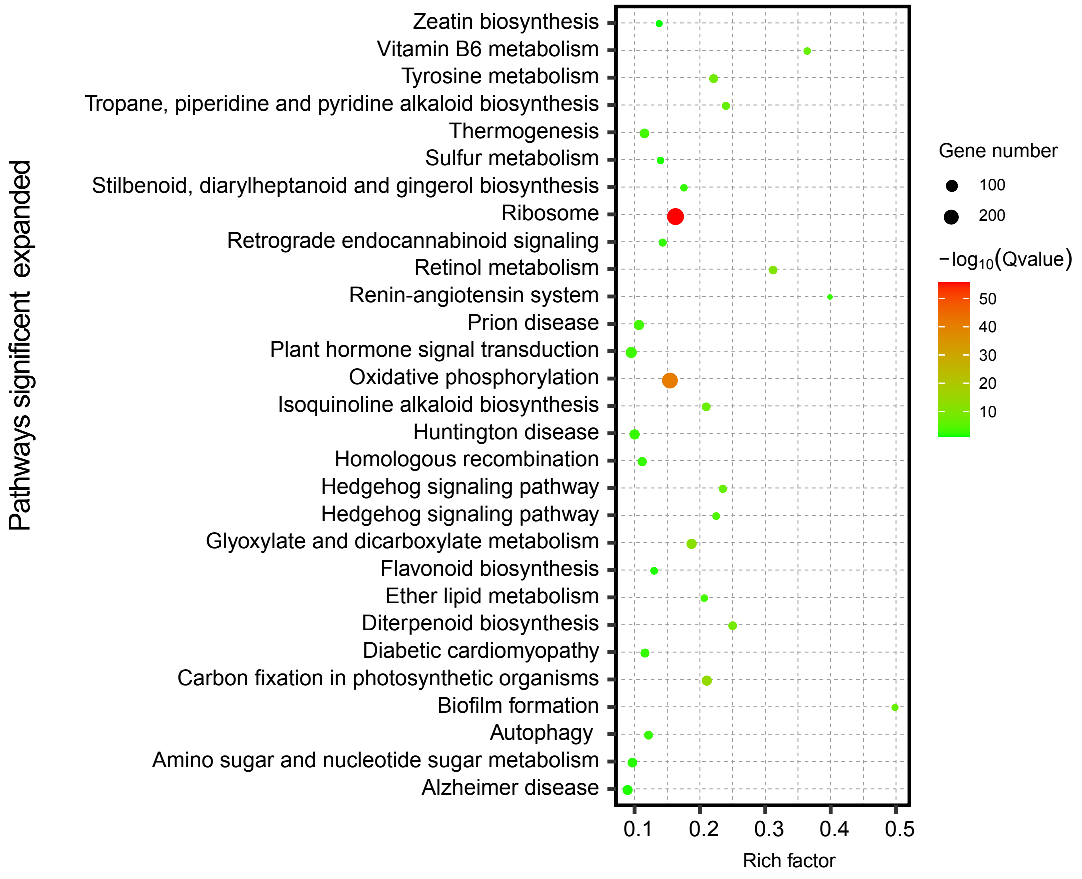

Figure S7. KEGG enrichment analysis of the expanded gene families in the *B. chinense* DC genome.
